# TRPA1 Channels Modify TRPV1-Mediated Current Responses in Dorsal Root Ganglion Neurons

**DOI:** 10.3389/fphys.2017.00272

**Published:** 2017-05-03

**Authors:** Takayoshi Masuoka, Makiko Kudo, Yuka Yamashita, Junko Yoshida, Noriko Imaizumi, Ikunobu Muramatsu, Matomo Nishio, Takaharu Ishibashi

**Affiliations:** Department of Pharmacology, School of Medicine, Kanazawa Medical UniversityUchinada, Japan

**Keywords:** TRPA1, TRPV1, dorsal root ganglion, intracellular calcium ion, membrane current

## Abstract

The transient receptor potential vanilloid 1 (TRPV1) channel is highly expressed in a subset of sensory neurons in the dorsal root ganglia (DRG) and trigeminal ganglia of experimental animals, responsible for nociception. Many researches have revealed that some TRPV1-positive neurons co-express the transient receptor potential ankyrin 1 (TRPA1) channel whose activities are closely modulated by TRPV1 channel. However, it is less investigated whether the activities of TRPV1 channel are modulated by the presence of TRPA1 channel in primary sensory neurons. This study clarified the difference in electrophysiological responses induced by TRPV1 channel activation between TRPA1-positive and TRPA1-negative DRG. TRPV1 and TRPA1 channel activations were evoked by capsaicin (1 μM), a TRPV1 agonist, and allyl isothiocyanate (AITC; 500 μM), a TRPA1 agonist, respectively. Capsaicin perfusion for 15 s caused a large inward current without a desensitization phase at a membrane potential of −70 mV in AITC-insensitive DRG (current density; 29.6 ± 5.6 pA/pF, time constant of decay; 12.8 ± 1.8 s). The capsaicin-induced currents in AITC-sensitive DRG had a small current density (12.7 ± 2.9 pA/pF) with a large time constant of decay (24.3 ± 5.4 s). In calcium imaging with Fura-2, the peak response by capsaicin was small and duration reaching the peak response was long in AITC-sensitive neurons. These electrophysiological differences were completely eliminated by HC-030031, a TRPA1 antagonist, in an extracellular solution or 10 mM EGTA, a Ca^2+^ chelator, in an internal solution. Capsaicin perfusion for 120 s desensitized the inward currents after a transient peak. The decay during capsaicin perfusion was notably slow in AITC-sensitive DRG; ratio of capsaicin-induced current 60 s after the treatment per the peak current in AITC-sensitive neurons (78 ± 9%) was larger than that in AITC-insensitive neurons (48 ± 5%). The capsaicin-induced current in the desensitization phase was attenuated by HC-030031 in AITC-insensitive DRG. These results indicate that (1) TRPV1-mediated currents in TRPA1-positive neurons characterize small current densities with slow decay, which is caused by TRPA1 channel activities and intracellular Ca^2+^ mobilization and (2) desensitization of TRPV1-mediated current in TRPA1-positive neurons is apparently slow, due to appending TRPA1-mediated current.

## Introuction

The transient receptor potential vanilloid 1 (TRPV1) channel is a polymodal sensor, which is sensitive to noxious heat, acidic pH, and irritant vanilloids, and is highly expressed in a subset of sensory neurons in the dorsal root ganglia (DRG) and trigeminal ganglia, predominantly contributing to nociception. The activation of TRPV1 channels, Ca^2+^-permeable cation channels, induces a large inward current, thereby elevating intracellular Ca^2+^ concentration because of the influx of extracellular Ca^2+^ in primary sensory neurons (Caterina et al., 1997). The depolarization of sensory neurons induced by the TRPV1-mediated current was shown to be transmitted as nociceptive information to the central nervous system (Caterina et al., 2000). The TRPV1-mediated current responses were found to be regulated by various receptors, such as bradykinin receptors (Tang et al., 2004), purinergic receptors (Stanchev et al., 2009), and glutamate receptors (Masuoka et al., 2015; Szteyn et al., 2015), thereby modulating nociception. In contrast, elevated intracellular Ca^2+^ concentration induced by TRPV1 channel activation affects the functions of nociceptors and ion channels, modulating nociception in primary sensory neurons. The Ca^2+^ influx feeds back on the TRPV1 channels, inhibiting their gating by binding to the intracellular Ca^2+^ sensor, calmodulin (Rosenbaum and Simon, 2007; Lau et al., 2012). The Ca^2+^ influx by TRPV1 directly activates anoctamin 1 chloride channels, potentiating TRPV1-mediated pain in DRG neurons (Takayama et al., 2015). TRPV1 channel activation with capsaicin inhibits the mechanosensitive Piezo1 and Piezo2 channels by depleting phosphatidylinositol 4,5-bisphosphate and its precursor phosphatidylinositol 4-phosphate from the plasma membrane through Ca^2+^-induced phospholipase Cδ activation in DRG neurons (Borbiro et al., 2015).

Some TRPV1-positive DRG neurons co-express the transient receptor potential ankyrin 1 (TRPA1) channel. Recent studies have revealed the functional and mechanical interactions between TRPA1 and TRPV1 channels, although the physiological significance of TRPA1 channels has been poorly understood in TRPV1-expressing sensory neurons. The first report showing the TRPA1-TRPV1 interaction described that the pharmacological desensitization of TRPA1-mediated responses is more pronounced in sensory neurons that lack TRPV1 than in neurons that express TRPV1 (Akopian et al., 2007). Thereafter, the probability of TRPA1 being open at negative holding potentials is reduced by TRPV1 channels because of the complex formation of TRPV1 and TRPA1 channels (Staruschenko et al., 2010). Tmem100 protein was recently reported to decrease the interaction between TRPV1 and TRPA1 channels in DRG neurons, potentiating TRPA1 channel properties (Weng et al., 2015). Therefore, TRPA1 channel-mediated responses are largely modulated by TRPV1 channels in primary sensory neurons. However, studies have scarcely investigated whether TRPV1 channel activities are modulated in the presence of TRPA1 channels. Here, we examined the differences in the kinetics of current responses induced by TRPV1 channel activation in TRPA1-positive and TRPA1-negative DRG neurons to elucidate the modulating effect of TRPA1 channels on TRPV1 channel activities.

## Materials and methods

### Animals

All mice were purchased from SLC (Shizuoka, Japan). The mice were housed in clear acrylic cages in a temperature-controlled room (25 ± 1°C) with a 12-h light/dark cycle (lights on from 07:00 to 19:00). All animal procedures were approved by the Ethics Committee of Kanazawa Medical University. The mice were humanely treated, according to the National Institutes of Health Guide for the Care and Use of Laboratory Animals and the Guiding Principles for the Care and Use of Laboratory Animals set by the Japanese Pharmacological Society.

### Preparation of primary cultures

Culture preparation was conducted as previously described (Masuoka et al., 2015, 2016). The C57BL/6J male and female mice (6- to 14-day-old) were anesthetized by the inhalation of isoflurane (Escain®; Mylan Inc., Cecil Township, PA, USA). DRG were rapidly dissected in ice-cold Ca^2+^/Mg^2+^-free artificial cerebrospinal fluid (Ca^2+^/Mg^2+^-free ACSF; 143.9 mM NaCl, 3.35 mM KCl, 21 mM NaHCO_3_, 9.9 mM glucose, 0.6 mM NaH_2_PO_4_) gassed with a mixture of 95% O_2_ and 5% CO_2_ (pH 7.4). Neurons were dissociated following treatment with 0.1% type II collagenase (240–265 U/mg; Worthington Biochemical Co., Lakewood, NJ, USA), 0.1% trypsin (Gibco, San Diego, CA, USA), and 0.01% DNase I (Sigma, St. Louis, MO, USA) in Ca^2+^/Mg^2+^-free ACSF and shaken (35 cycle/min) in a water bath at 37°C for 30 min. Cells were gently triturated in Dulbecco's modified Eagle medium (Sigma) containing 10% horse serum (Gibco), 5% fetal calf serum (Gibco), and 1% penicillin–streptomycin (Wako, Osaka, Japan). Dispersed cells were passed through a 100-μm cell strainer (BD Biosciences, San Jose, CA, USA), and the filtered cells were seeded on glass coverslips (13 mm in diameter) coated with poly-L-lysine (Matsunami Glass Ind., Osaka, Japan).

### Whole-cell patch clamp recording

Cultured neurons were plated onto coverslips, transferred to the recording chamber, and superfused with ACSF (138.6 mM NaCl, 3.35 mM KCl, 21 mM NaHCO_3_, 9.9 mM glucose, 0.6 mM NaH_2_PO_4_, 2.5 mM CaCl_2_, and 1 mM MgCl_2_). Neurons were visually identified using a 60 × microscope objective (DIAPHOT300; Nikon, Tokyo, Japan). Whole-cell recording were performed from small and medium size of neurons (<25 μm diameter). Pipettes for whole-cell recordings were made from borosilicate glass capillaries (1.5-mm outer diameter; World Precision Instruments Inc., Sarasota, FL, USA). Patch pipettes (4–6 MΩ) were filled with an internal solution containing 120 mM KCH_3_SO_3_, 5 mM KCl, 0.1 mM K-ethylene glycol-bis(β-aminoethyl ether)-N,N,N′,N′-tetraacetic acid (EGTA), 5 mM Na-4-(2-hydroxyethyl)-1-piperazineethanesulfonic acid, 3 mM Mg-adenosine triphosphate, and 0.4 mM Na-guanosine triphosphate (pH 7.4). Series resistance was 8–20 MΩ, which was monitored throughout the recording. Membrane currents were recorded in a whole-cell configuration using an Axopatch-1D amplifier and pCLAMP 10 software (Axon Instruments, Foster City, CA, USA), digitized, and stored on a computer disk for offline analysis. Current responses mediated by TRPV1 and TRPA1 were induced through 0.03–1 μM capsaicin (a TRPV1 agonist) perfusion for 15 or 120 s and 500 μM AITC (a TRPA1 agonist) perfusion for 30 s, respectively. Capsaicin responses were recorded >5 min after establishing whole-cell configuration. AITC-induced responses were observed >5 min after capsaicin perfusion. The extracellular solution was perfused at 2 mL/min. To clarify contribution of TRPA1 channels in capsaicin-induced current, HC-030031 (a TRPA1 antagonist) was dissolved in ACSF with or without capsaicin and was perfused. Temperature in recording chamber was maintained at 30.0 ± 1.0°C with in-line solution heaters (SF-28; Warner Instruments, Hamden, CT, USA) and temperature controller (TC-324C; Warner Instruments).

### Calcium imaging

Changes in intracellular calcium were measured with a fluorescent calcium indicator, as described previously (Masuoka et al., 2015, 2016). For microscopic fluorometric measurement, cultured DRG neuronal cells were washed twice with ACSF and incubated for 45 min in the CO_2_ incubator (37 ± 2°C) in a solution of 3 μM of Fura-2-acetoxymethyl ester (Fura-2 AM; Dojindo Laboratories, Kumamoto, Japan) and 0.005% Cremophor EL (Sigma). After incubation, cells were washed in ACSF for 30 min and culture dishes were placed on the stage of an inverted microscope (ECLIPSE TE 300, Nikon, Tokyo, Japan) equipped with a 20 × S-fluor objective. Fluorescence images were recorded and analyzed using a video image analysis system (ARGUS/HiSCA, Hamamatsu Photonics, Hamamatsu, Japan). Experimental agents were dissolved in ACSF and delivered by continuous perfusion in the recording chamber (2 mL/min) with a peristaltic pump. Capsaicin (1 mμM), AITC (500 μM) and KCl (50 mM) were respectively perfused for 15, 30, and 30 s in this order. Image pairs were captured at 5 s (beginning 5 min) or 10 s intervals. Fura-2 fluorescence was recorded at an emission wavelength of 510 nm by exciting Fura-2 at 340 and 380 nm. The 340–380 nm fluorescence ratio (F340/F380) was used as a parameter of intracellular calcium concentration.

### Drugs

Capsaicin and allyl isothiocyanate (AITC) were obtained from Sigma-Aldrich (St. Louis, MO, USA); 2-(1,3-dimethyl-2,6-dioxo-1,2,3,6-tetrahydro-7H-purin-7-yl)-N-(4-isopropylphenyl)acetamide (HC-030031) was obtained from Abcam (Cambridge, United Kingdom). The stock solution of capsaicin and AITC were dissolved in ethanol at the concentration of 10 mM and 2 M, respectively. HC-030031 was dissolved in DMSO at 100 mM. The stock solutions were diluted with ACSF just before the experiment. The working solutions were made not exceeding 0.03% ethanol, because 0.1–3% ethanol facilitates TRPV1 channels activity induced by capsaicin (Trevisani et al., 2002).

### Statistical analysis

Data are expressed as mean ± standard error of the mean (SEM), and “n” represents the number of cells examined. Data were analyzed using the SigmaPlot 13.0 software (Systat Software Inc., San Jose, CA, USA). Data were assessed using the unpaired *t*-test. A *P* value of < 0.05 was considered significant.

## Results

**Figure 1:**
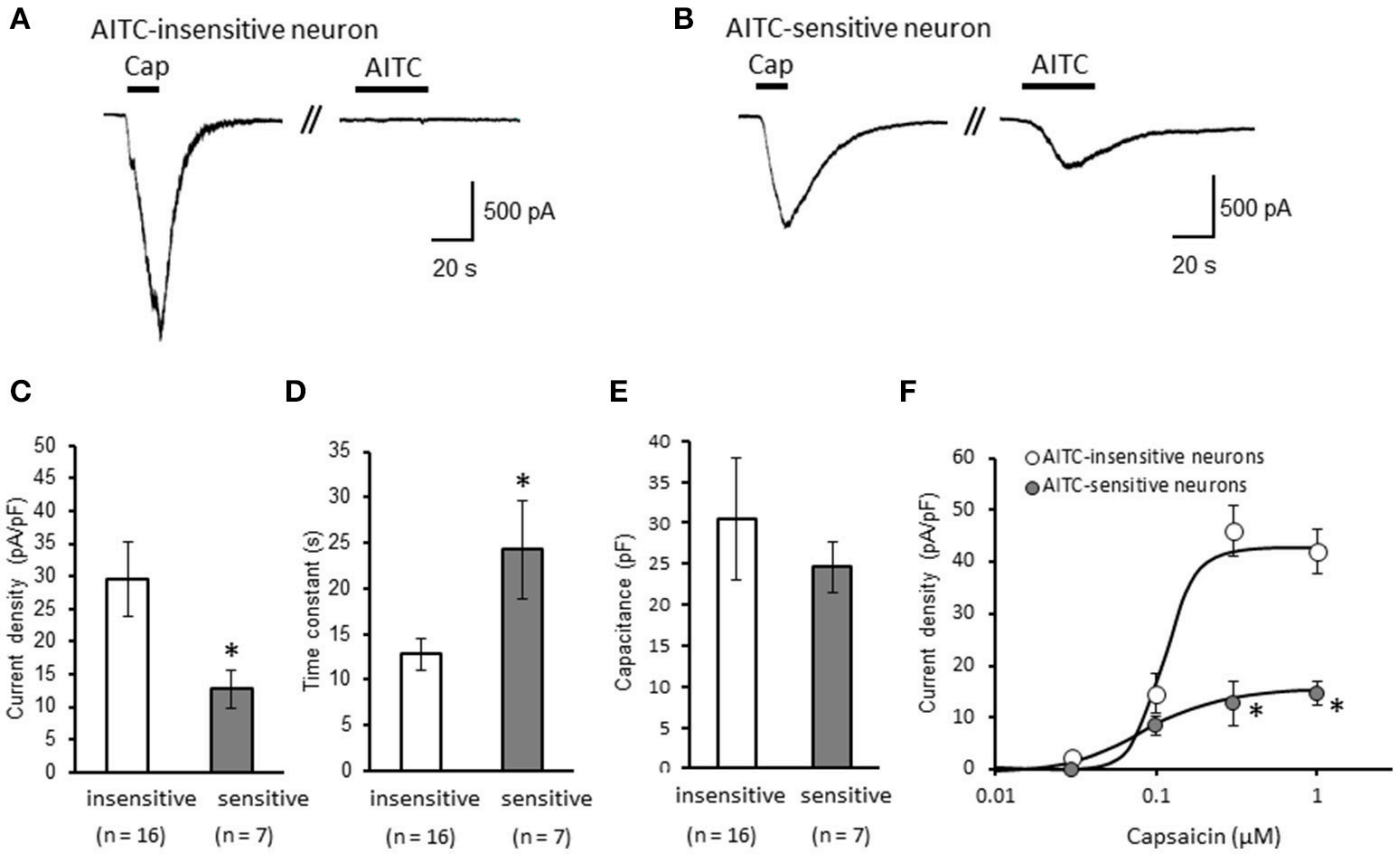
**Differences in capsaicin-induced current between AITC-insensitive and AITC-sensitive DRG neurons. (A,B)** Representative traces of current response induced by 1 μM capsaicin (cap) perfusion for 15 s and 500 μM AITC perfusion for 30 s in AITC-insensitive **(A)** and AITC-sensitive **(B)** DRG neurons. The currents were recorded with a potassium methanesulfonate internal solution at membrane potentials of −70 mV. **(C–E)** The differences in the capsaicin-induced current density **(C)**, time constant of decay **(D)**, and membrane capacitance **(E)** between AITC-insensitive DRG neurons (open columns) and AITC-sensitive DRG neurons (gray columns) are shown. **(F)** Concentration–response curves to capsaicin in AITC-insensitive and AITC-sensitive DRG neurons. Each column and vertical bar represent the mean ± standard error of the mean (SEM). ^*^P < 0.05 by unpaired *t*-test.

**Figure 2:**
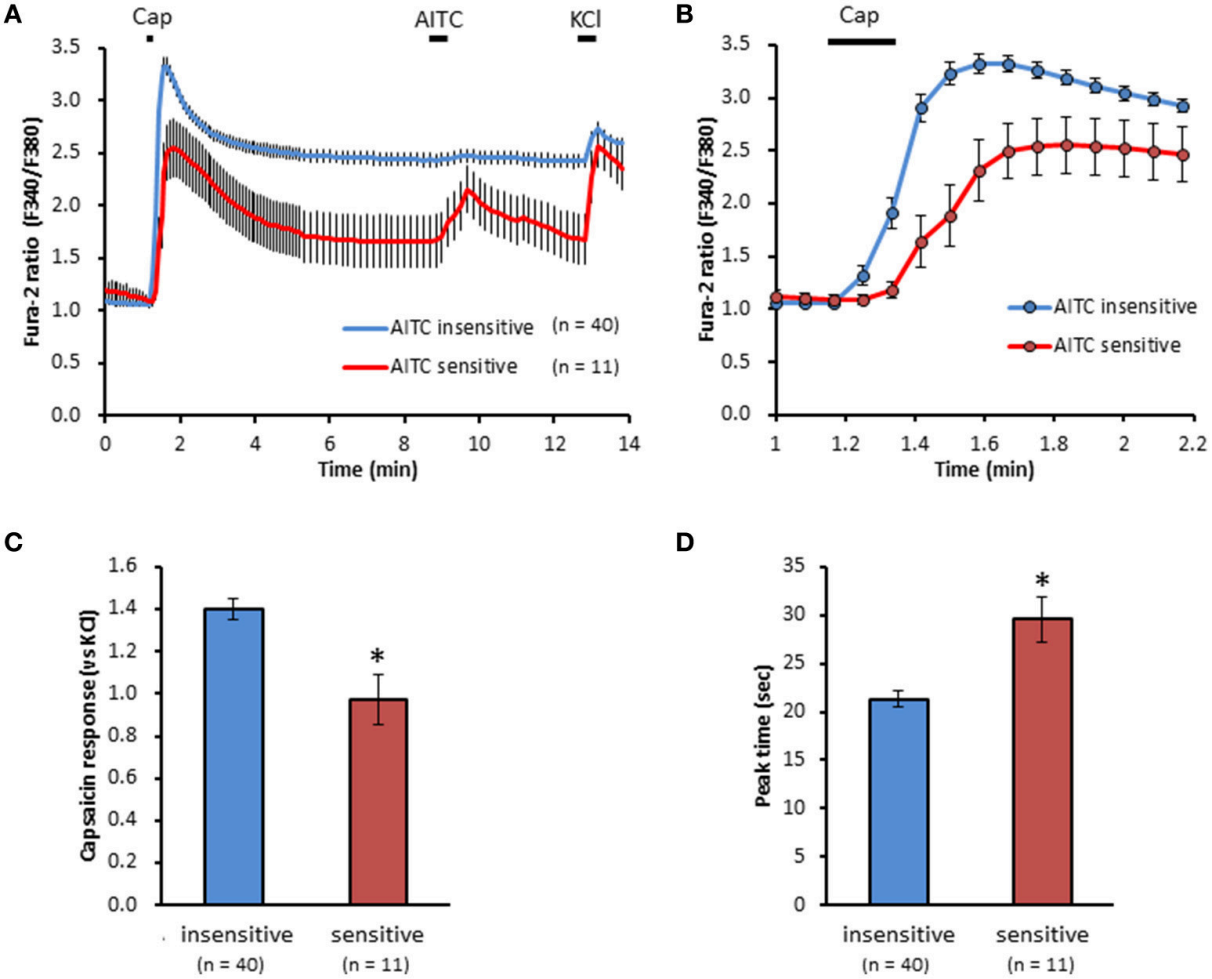
**Differences in capsaicin-induced intracellular calcium elevation between AITC-insensitive and AITC-sensitive DRG neurons. (A)** Time course of F340/F380 ratio before and after the perfusion of capsaicin (cap, 1 μM) AITC (500 μM) and KCl (50 mM) using Fura-2 AM dye. Horizontal bars represent periods of capsaicin, AITC and KCl perfusion. **(B)** Changes in F340/F380 ratio with a widespread expression of x axis between time 1 and 2.2 min of **(A)**. The difference in peak response **(C)** and time reaching peak response **(D)** on capsaicin-induced elevation of intracellular calcium in DRG neurons. Data presented as mean ± SEM. ^*^P < 0.05 against AITC-sensitive group.

**Figure 3:**
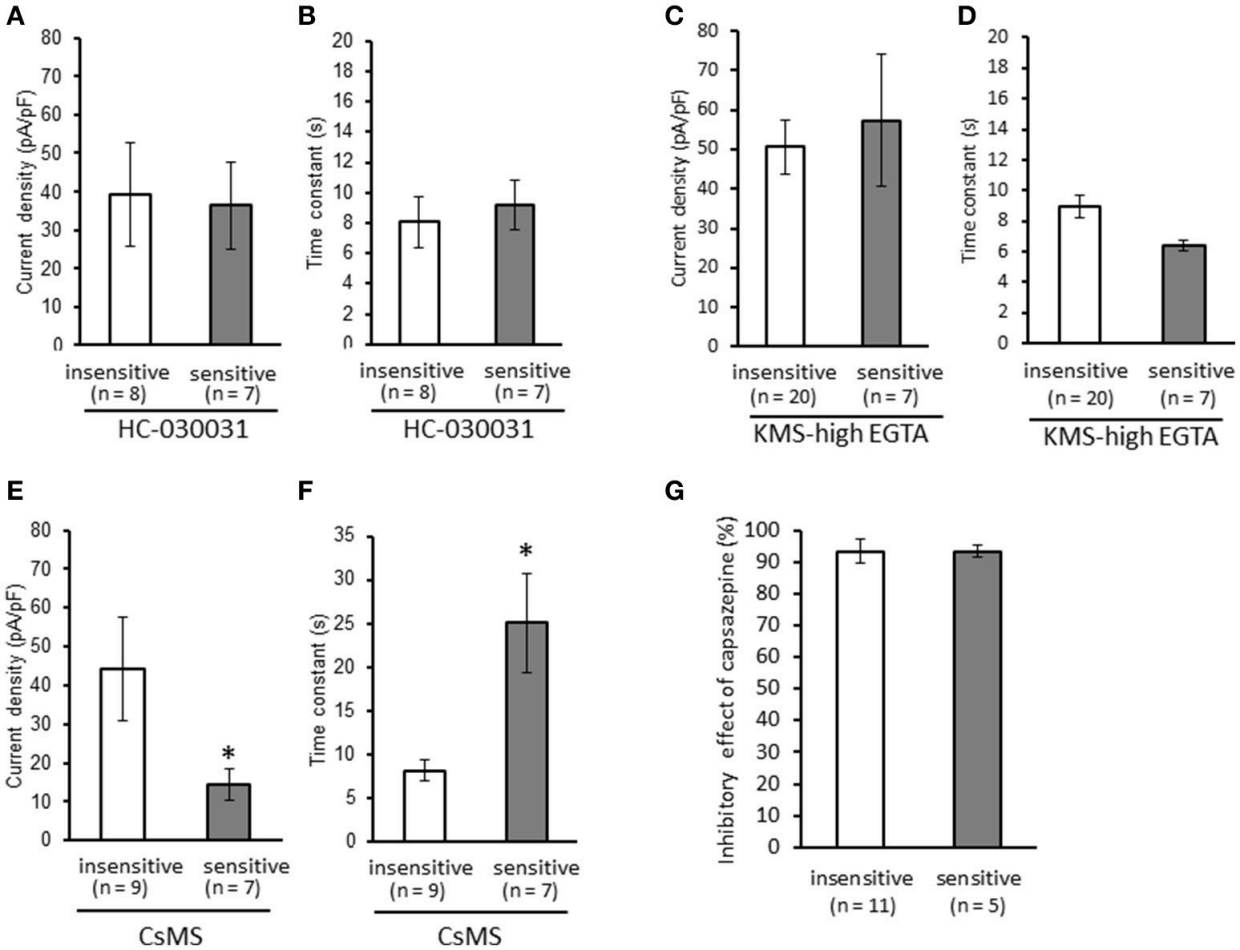
**Contributions of TRPA1 channels, intracellular Ca^2+^ concentration, and potassium channels to the differences in kinetics of capsaicin-induced currents between AITC-insensitive and AITC-sensitive DRG neurons**. Capsaicin-induced currents were recorded **(A,B)** in the presence of HC-030031 (5 μM, a TRPA1 antagonist), **(C,D)** with intracellular solutions containing a high EGTA concentration (10 mM, a Ca^2+^ chelator), and **(E,F)** with intracellular solutions containing cesium methanesulfonate (CsMS) instead of potassium methanesulfonate (KMS) to block potassium channels. The differences in the current density **(A,C,E)** and time constant of decay **(B,D,F)** are shown in each recording condition. **(G)** Effect of capsazepine on capsaicin-induced current in AITC-sensitive and AITC-insensitive neurons. Each column and vertical bar represent the mean ± SEM. ^*^P < 0.05 by unpaired *t*-test.

We clarified the difference in kinetics of current responses to TRPV1 channel activation between TRPA1-negative and TRPA1-positive DRG neurons using whole-cell recording. TRPV1 channel activation was evoked by the perfusion of 1 μM capsaicin, a TRPV1 agonist. To identify TRPA1-positive and TRPA1-negative neurons, AITC (a TRPA1 agonist) were perfused after recording of capsaicin responses in this study. Some studies clarified that high concentration (1 mM and over) of AITC activates porcine, mouse and human TRPV1 as well as TRPA1 (Ohta et al., 2007; Everaerts et al., 2011; Gees et al., 2013). Therefore, TRPA1-negative and TRPA1-positive DRG neurons were distinguished by responsiveness to perfusion of 500 μM AITC for 30 s, >5 min after capsaicin perfusion. In this condition, peak current were observed at 15–25 s after starting AITC perfusion (Figure 1B), which were completely blocked by 10 μM HC-030031, a TRPA1 antagonist. A few recording showed very small currents (<100 pA) without peak response before washout of AITC; they were probably TRPV1-mediated current by AITC. In this study, we excluded these data to accurately identify TRPA-positive and TRPA1-negative neurons. Capsaicin perfusion for 15 s caused a large inward current without a desensitization phase at a membrane potential of −70 mV (Figures 1A,B). The capsaicin-induced currents in AITC-sensitive DRG neurons had a small current density (Figure 1C) with a large time constant of decay (Figure 1D). Membrane capacitance in recording neurons demonstrated no significant difference between the two groups (Figure 1E). The capsaicin-induced current density increased in a concentration-dependent manner (Figure 1F). The maximum response was significantly smaller in AITC-sensitive DRG neurons (14.9 ± 4.7 pA/pF) than in AITC-insensitive ones (43.8 ± 4.3 pA/pF); furthermore, EC_50_ showed no discernible difference between the two groups (0.088 ± 0.008 and 0.104 ± 0.024 μM, respectively). Fluorescence calcium imaging is useful to analyze TRPV1 channels activities, because TRPV1 channels are high-conductance Ca^2+^-permeable TRP channels. Intracellular calcium mobilizations by capsaicin were examined with Fura-2 (Figure 2). Perfusion of 1 μM capsaicin increased the intracellular calcium concentration in a subset of DRG neurons. Elevations of intracellular calcium concentration in AITC-sensitive neurons were significantly smaller than that in AITC-insensitive neurons (Figures 2B,C). In addition, duration reaching peak responses after starting the perfusion was significantly longer in AITC-sensitive neurons (Figures 2B,D). These results confirmed that small current densities and slow decay of capsaicin-induced current in AITC-sensitive neurons. We examined the possible contributions of TRPA1 channels, intracellular Ca^2+^ concentration, and potassium channels to the difference in kinetics of TRPV1-mediated current between AITC-sensitive DRG neurons and AITC-insensitive ones (Figures 1C,D). Capsaicin responses under presence of HC-030031 were examined more than 5 min after perfusion of HC-030031. The differences in current density and time constant of decay were completely eliminated in the presence of 5 μM HC-030031, a TRPA1 antagonist, in the extracellular solution (Figures 3A,B). To explore the contributions of intracellular Ca^2+^ to the different kinetics of capsaicin-induced currents between AITC-insensitive and AITC-sensitive DRG neurons, we recorded the TRPV1-mediated current induced by capsaicin with intracellular solutions containing 10 mM EGTA, a Ca^2+^ chelator. The differences in the current density and time constant of decay (Figures 1C,D) completely disappeared when a high EGTA concentration was added in the internal solution (Figures 3C,D). In contrast, although potassium in the internal solution was replaced by Cs^+^ to block the potassium channels, TRPV1-mediated currents in AITC-sensitive DRG neurons were significantly smaller with a larger time constant of decay than those in AITC-insensitive DRG neurons (Figures 3E,F). Presence of 10 μM capsazepine, a TRPV1 antagonist, strongly inhibited capsaicin-induced current in both AITC-insensitive and AITC-sensitive neurons, which has no difference between two groups (Figure 3G). These results suggest that the different kinetics of capsaicin-induced current between AITC-insensitive and AITC-sensitive DRG neurons are caused by TRPA1 channels activities and the dynamics of intracellular Ca^2+^ concentration. To elucidate the presence of TRPA1 current in the basal condition in AITC-sensitive neurons, the effect of HC-030031 was examined before the application of capsaicin and AITC. The perfusion of HC-030031 (5 μM) for 60 s slightly but significantly attenuated the basal inward current in capsaicin- and AITC-sensitive neurons (−17.3 ± 4.4 pA, *n* = 5, *P* < 0.05), although it had no effect in capsaicin-sensitive and AITC-insensitive neurons (2.0 ± 2.7 pA, *n* = 13). This implied that a tonic TRPA1 current exists in AITC-sensitive neurons in the absence of exogenous TRPV1 and TRPA1 agonists.

**Figure 4:**
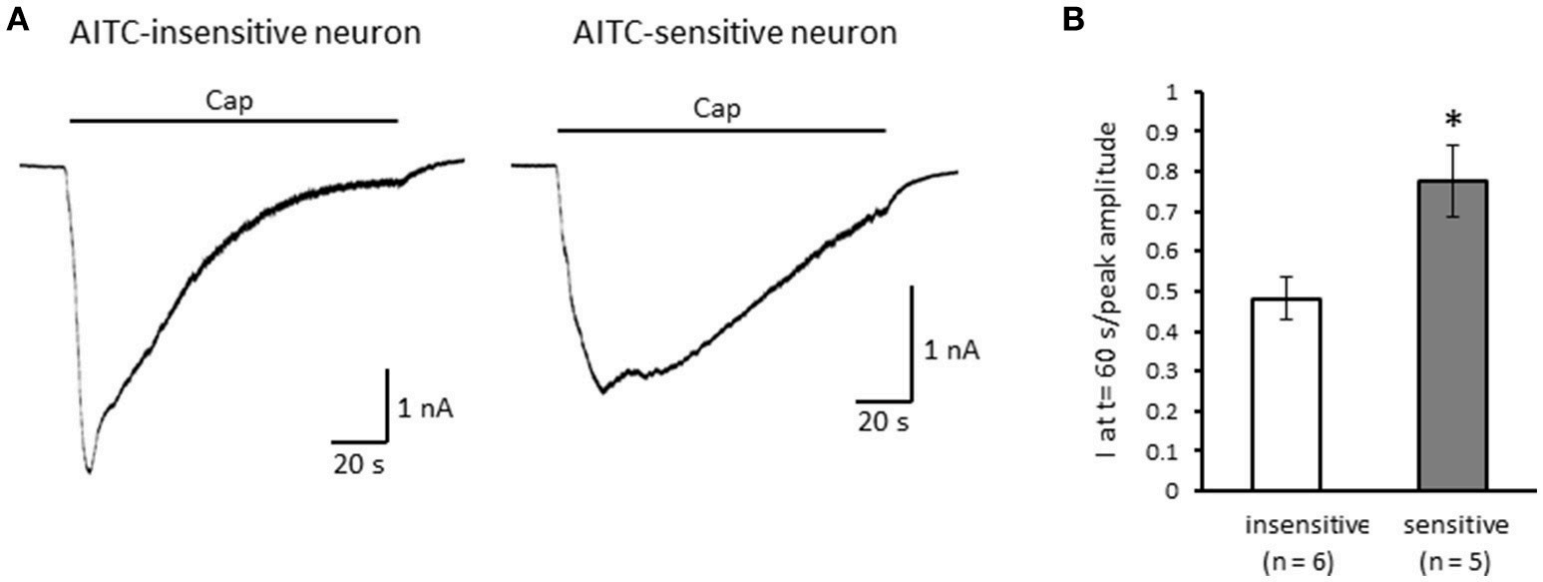
**The differences in desensitization of currents induced by persistent perfusion of capsaicin. (A)** Representative traces of current response induced by 1 μM capsaicin (cap) perfusion for 120 s in AITC-insensitive (left) and AITC-sensitive (right) DRG neurons. **(B)** The difference in the decay of capsaicin-induced current 60 s after initiating the perfusion between AITC-insensitive DRG neurons (open column) and AITC-sensitive DRG neurons (gray column). Each column and vertical bar represent the mean ± SEM. ^*^P < 0.05 by unpaired *t*-test.

**Figure 5:**
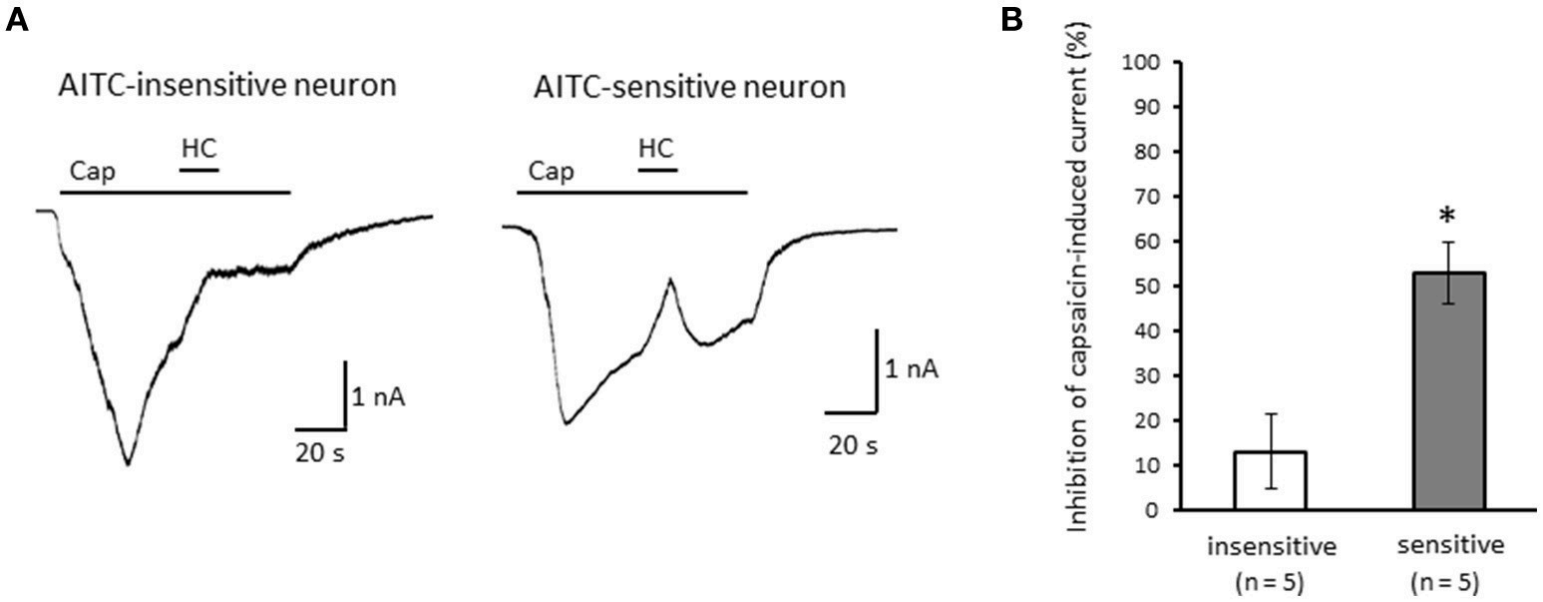
**Effects of HC-030031 on capsaicin-induced current in the desensitization phase. (A)** Representative traces of capsaicin (cap)-induced current during 2 μM HC-030031 (HC) perfusion in AITC-insensitive (left) and AITC-sensitive (right) DRG neurons. **(B)** Inhibitory effects of HC-030031 on capsaicin-induced current in AITC-insensitive DRG neurons (open column) and in AITC-sensitive DRG neurons (gray column). Each column and vertical bar represent the mean ± SEM. ^*^P < 0.05 by unpaired *t*-test.

**Figure 6:**
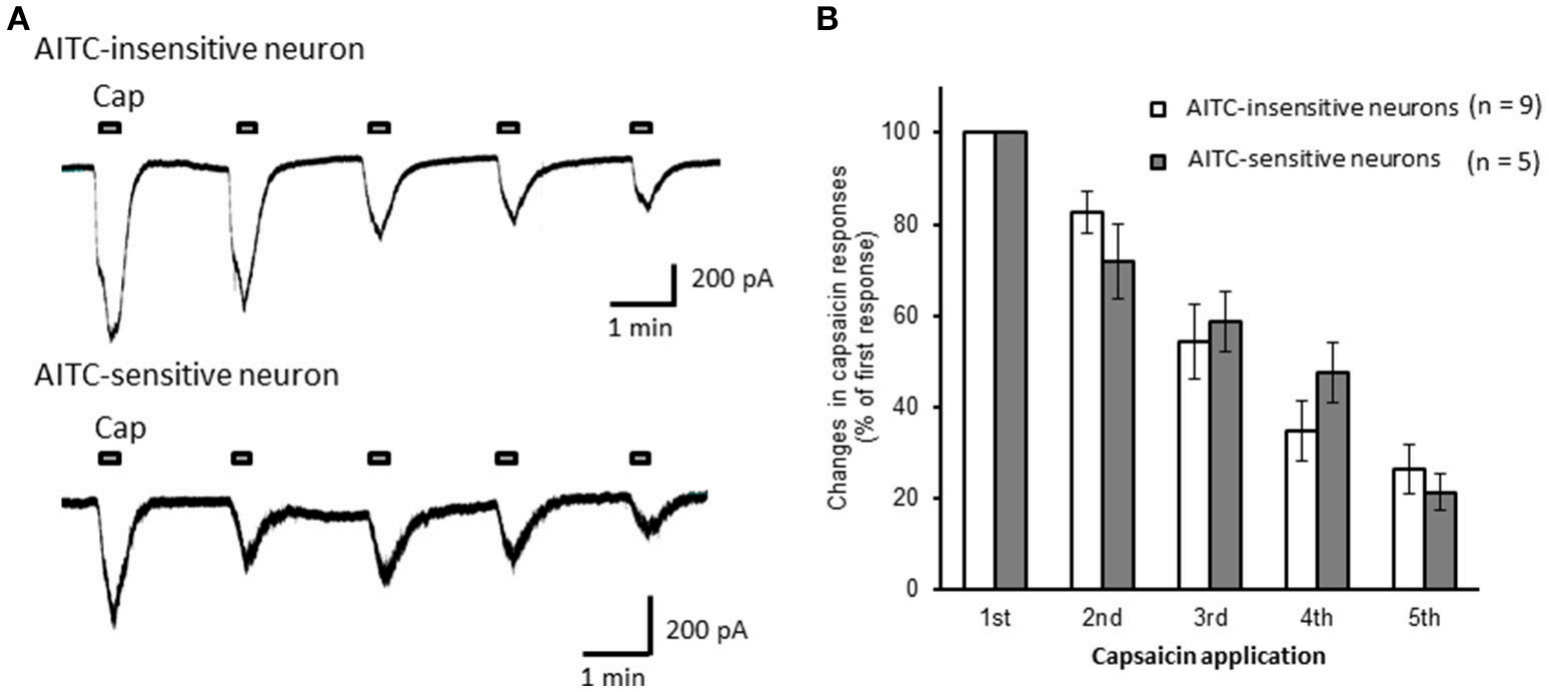
**The desensitization of capsaicin-induced currents by repeated application in AITC-insensitive and AITC-sensitive DRG neurons**. **(A)** Representative traces of current induced by repeated capsaicin (cap) perfusion in AITC-insensitive (upper) and AITC-sensitive (lower) DRG neurons. **(B)** Comparison of desensitization of capsaicin-induced current between AITC-insensitive DRG neurons (open columns) and AITC-sensitive DRG neurons (gray columns). Each column and vertical bar represent the mean ± SEM.

Next, we examined the difference in the desensitization of current induced by TRPV1 channel activation between AITC-sensitive and AITC-insensitive DRG neurons. When 1 μM capsaicin was perfused for 120 s (Figure 4A), the transient peak current was observed 15–30 s after the perfusion in all neurons, which then gradually desensitized the inward currents. As the decay of the capsaicin-induced current was difficult to fit with a logarithmic curve in many AITC-sensitive DRG neurons, we calculated the ratio of current 60 s after treatment of capsaicin per peak current as an alternative measure. The ratio of the current was significantly larger in AITC-sensitive DRG neurons than in AITC-insensitive DRG neurons, indicating that the decay of the capsaicin-induced current in the desensitization phase was significantly slower in TRPA1-positive DRG neurons than in TRPA1-negative DRG neurons (Figure 4B). The effects of HC-030031 were subsequently examined. HC-030031 perfusion significantly inhibited capsaicin-induced currents in AITC-sensitive DRG neurons, which was absent in AITC-insensitive DRG neurons (Figure 5A). The inhibitory effect of HC-030031 was significantly larger in AITC-sensitive DRG neurons than in AITC-insensitive DRG ones (Figure 5B). Therefore, TRPA1-mediated current is contained in the capsaicin-induced current in the desensitization phase in AITC-sensitive DRG neurons. To clarify the difference in the desensitization of the TRPV1-mediated current by repeated stimulation between AITC-sensitive and AITC-insensitive DRG neurons, 1 μM capsaicin perfusion for 15 s was repeated five times. Repeated short-term capsaicin perfusion gradually desensitized capsaicin-induced current responses (Figure 6A), and no difference was found between the two groups (Figure 6B).

## Discussion

We clarified that the kinetics of TRPV1-mediated current induced by capsaicin differed depending on the coexistence of TRPA1 channels in DRG neurons. First, the current densities were significantly smaller in AITC-sensitive DRG neurons than in AITC-insensitive DRG neurons. The difference disappeared in the presence of a high EGTA concentration in internal solutions and in the presence of HC-030031 in extracellular solutions. Therefore, TRPA1 channels suppress TRPV1 channel activity, possibly by regulating the basal intracellular Ca^2+^ concentration. TRPA1 channels are activated by exogenous irritants, including mustard oil, allicin, acrolein (Bautista et al., 2006), and alkaline pH (Fujita et al., 2008), and by endogenous substances, including hydrogen peroxide, nitric oxide, hydrogen sulfide, oxidized lipids, and general long-chain polyunsatulated fatty acids (Andersson et al., 2008, 2012; Cavanaugh et al., 2008; Takahashi et al., 2008; Motter and Ahern, 2012). Basal inward currents in AITC-sensitive neurons were slightly attenuated by a TRPA1 antagonist, which implies that a tonic TRPA1 current exists in DRG neurons. Therefore, the spontaneous TRPA1 channel activity possibly induced by endogenous substances inhibits proximate TRPV1 channels mediated by Ca^2+^ elevation. Intracellular calcium concentrations regulate the activities of protein kinase A, protein kinase C, Ca^2+^/Calmodulin-dependent protein kinase II, and calcineurin that regulate TRPV1 channels activities through phosphorylation or dephosphorylation of several amino acid residues in TRPV1 channels, such as Ser502, Thr704, and Ser800 (Rosenbaum and Simon, 2007). The dephosphorylation of TRPV1 by calcineuline that activated by weak intracellular calcium elevation desensitizes TRPV1 channels activities by vanilloid stimulation, capsaicin (Jung et al., 2004). The balance of phosphorylation and dephosphorylation of TRPV1 under presence of TRPA1 possibly contributes to the mechanism of small current densities of TRPV1 channels. Second, the latencies of decay in capsaicin-induced currents were significantly longer in AITC-sensitive DRG neurons than in AITC-insensitive DRG neurons, which was blocked by an internal solution containing a high EGTA concentration and in the presence of HC-030031 in an extracellular solution. In addition, desensitization during persistent capsaicin perfusion was notably slower in AITC-sensitive DRG neurons than in AITC-insensitive DRG neurons. The capsaicin-induced current in the desensitization phase was attenuated by HC-030031 in AITC-sensitive DRG neurons. Therefore, it seems that TRPA1-mediated current are evoked after activation of TRPV1 channels by capsaicin. TRPA1 channels are activated by intracellular Ca^2+^ binding to the EF-hand domain (Zurborg et al., 2007; Paulsen et al., 2015). TRPA1 channels are directly activated by endogenous substance produced by intracellular calcium elevation. For instance, nitric oxide is synthesized by nitric oxide synthase (NOS) whose activation is essential for increase in intracellular Ca^2+^ bringing about calmodulin binding (Förstermann and Sessa, 2012), leading to activates TRPA1 channels through nitrosylation of Cys421, Cys641, and Cys665 (Takahashi et al., 2008). Actually, the neuronal NOS predominantly expresses in small and medium size of DRG neurons (Terenghi et al., 1993; Kolesár et al., 2016). Considering the abovementioned reports, TRPV1 channels may directly and/or indirectly activate TRPA1 channels by elevating intracellular Ca^2+^ concentration in DRG neurons, which seems to underlie slow decay of capsaicin-induced current after the brief perfusion and slow desensitization of capsaicin-induced current during the long-term perfusion in AITC–sensitive neurons.

Small peak currents induced by TRPV1 channel activation in TRPA1-positive DRG neurons might implicate low excitability for noxious stimuli in sensory neurons that co-express TRPV1 and TRPA1 channels under normal conditions. In contrast, the slow desensitization of TRPV1-mediated current in TRPA1-positive DRG neurons might enable nociceptive stimuli to persistently excite primary sensory neurons. The tumor necrosis factor-alpha released from mast cells, lymphocytes, and skin keratinocytes during inflammation increases the cotrafficking of TRPA1/TRPV1 in trigeminal ganglion, which is involved in hypersensation in an inflammatory disorder (Meng et al., 2016). A previous study revealed that long-term treatment with glutamate, an endogenous pain modulator and inducer of inflammation, drastically increased TRPV1-mediated currents induced by capsaicin in TRPA1-expressing DRG neurons (Masuoka et al., 2016). The facilitation of TRPV1-mediated currents in TRPA1-positive DRG neurons produced heat hyperalgesia in mice (Masuoka et al., 2016). Therefore, TRPV1-mediated electrophysiological responses in TRPA1-expressing sensory neurons might be related to the molecular basis of nociception in chronic abnormal pain induced by inflammation. Recent studies clarified that sensory neurons that express TRPA1 regulate inflammation and pruritogen responses (Wilson et al., 2011; Liu et al., 2013; Cevikbas et al., 2014). For instance, TRPA1 is required for Mas-related G protein-coupled receptor-mediated signaling, which is activated by mast cell mediators and promotes histamine-independent itch (Wilson et al., 2011). Interleukin-31 (IL-31), T helper cell type 2- derived cytokine, activates a small subpopulation of primary sensory neurons expressing TRPV1, and TRPA1 through IL-31 receptor, and produced inflammatory and lymphoma-associated itch (Cevikbas et al., 2014). Therefore, TRPV1 channel activation in DRG neurons that co-express TRPA1 channels might contribute to the itch response. Our findings might help in understanding the characteristics and molecular mechanisms of itch often accompanied by pain.

## Conclusion

TRPV1 mediated-currents in TRPA1-positive neurons are characterized small densities with slow decay, which is caused by TRPA1 channels activation and intracellular calcium mobilization. In addition, TRPV1-mediated current in TRPA1-possitive neurons slowly desensitize, due to appending TRPA1-mediated current.

## Author contributions

TM designed and performed the research, analyzed the data, and wrote the paper. MK and YY performed research and analyzed data. JY, NI, IM, MN, and TI wrote and revised the paper.

## Funding

This work was supported by Grant for Promoted Research from Kanazawa Medical University (Grant No. S2015-8, S2016-5), by JSPS KAKENHI grants (Grant Nos. 26460348, 15K08250, 16K19023, and 17K16992), and by the Smoking Research Foundation of Japan.

### Conflict of interest statement

The authors declare that the research was conducted in the absence of any commercial or financial relationships that could be construed as a potential conflict of interest.

## References

[B1] Akopian A. N. Ruparel N. B. Jeske N. A. Hargreaves K. M. (2007). Transient receptor potential TRPA1 channel desensitization in sensory neurons is agonist dependent and regulated by TRPV1-directed internalization. J. Physiol. 583, 175–193. 10.1113/jphysiol.2007.133231 17584831 PMC2277224

[B2] Andersson D. A. Gentry C. Bevan S. (2012). TRPA1 has a key role in the somatic pro-nociceptive actions of hydrogen sulfide. PLoS ONE 7:e46917. 10.1371/journal.pone.0046917 23071662 PMC3469557

[B3] Andersson D. A. Gentry C. Moss S. Bevan S. (2008). Transient receptor potential A1 is a sensory receptor for multiple products of oxidative stress. J. Neurosci. 28, 2485–2494. 10.1523/JNEUROSCI.5369-07.2008 18322093 PMC2709206

[B4] Bautista D. M. Jordt S. E. Nikai T. Tsuruda P. R. Read A. J. Poblete J.. (2006). TRPA1 mediates the inflammatory actions of environmental irritants and proalgesic agents. Cell 124, 1269–1282. 10.1016/j.cell.2006.02.023 16564016

[B5] Borbiro I. Badheka D. Rohacs T. (2015). Activation of TRPV1 channels inhibits mechanosensitive Piezo channel activity by depleting membrane phosphoinositides. Sci. Signal. 8, ra15. 10.1126/scisignal.2005667 25670203 PMC4527171

[B6] Caterina M. J. Leffler A. Malmberg A. B. Martin W. J. Trafton J. Petersen-Zeitz K. R.. (2000). Impaired nociception and pain sensation in mice lacking the capsaicin receptor. Science 288, 306–313. 10.1126/science.288.5464.306 10764638

[B7] Caterina M. J. Schumacher M. A. Tominaga M. Rosen T. A. Levine J. D. Julius D. (1997). The capsaicin receptor: a heat-activated ion channel in the pain pathway. Nature 389, 816–824. 10.1038/39807 9349813

[B8] Cavanaugh E. J. Simkin D. Kim D. (2008). Activation of transient receptor potential A1 channels by mustard oil, tetrahydrocannabinol and Ca^2+^ reveals different functional channel states. Neuroscience 154, 1467–1476. 10.1016/j.neuroscience.2008.04.048 18515013

[B9] Cevikbas F. Wang X. Akiyama T. Kempkes C. Savinko T. Antal A.. (2014). A sensory neuron-expressed IL-31 receptor mediates T helper cell-dependent itch: involvement of TRPV1 and TRPA1. J. Allergy Clin. Immunol. 133, 448–460. 10.1016/j.jaci.2013.10.048 24373353 PMC3960328

[B10] Everaerts W. Gees M. Alpizar Y. A. Farre R. Leten C. Apetrei A.. (2011). The capsaicin receptor TRPV1 is a crucial mediator of the noxious effects of mustard oil. Curr. Biol. 21, 316–321. 10.1016/j.cub.2011.01.031 21315593

[B11] Förstermann U. Sessa W. C. (2012). Nitric oxide synthases: regulation and function. Eur. Heart J. 33, 829–837. 10.1093/eurheartj/ehr304 21890489 PMC3345541

[B12] Fujita F. Uchida K. Moriyama T. Shima A. Shibasaki K. Inada H.. (2008). Intracellular alkalization causes pain sensation through activation of TRPA1 in mice. J. Clin. Invest. 118, 4049–4057. 10.1172/JCI35957 19033673 PMC2582441

[B13] Gees M. Alpizar Y. A. Boonen B. Sanchez A. Everaerts W. Segal A.. (2013). Mechanisms of transient receptor potential vanilloid 1 activation and sensitization by allyl isothiocyanate. Mol. Pharmacol. 84, 325–334. 10.1124/mol.113.085548 23757176

[B14] Jung J. Shin J. S. Lee S. Y. Hwang S. W. Koo J. Cho H.. (2004). Phosphorylation of vanilloid receptor 1 by Ca^2+^/calmodulin-dependent kinase II regulates its vanilloid binding. J. Biol. Chem. 279, 7048–7054. 10.1074/jbc.M311448200 14630912

[B15] Kolesár D. Kolesárová M. Kyselovič J. (2016). Distribution pattern of dorsal root ganglion neurons synthesizing nitric oxide synthase in different animal species. Can. J. Physiol. Pharmacol. 24, 1–5. 10.1139/cjpp-2016-0294 28103057

[B16] Lau S. Y. Procko E. Gaudet R. (2012). Distinct properties of Ca^2+^-calmodulin binding to N- and C-terminal regulatory regions of the TRPV1 channel. J. Gen. Physiol. 140, 541–555. 10.1085/jgp.201210810 23109716 PMC3483115

[B17] Liu B. Escalera J. Balakrishna S. Fan L. Caceres A. I. Robinson E.. (2013). TRPA1 controls inflammation and pruritogen responses in allergic contact dermatitis. FASEB J. 27, 3549–3563. 10.1096/fj.13-229948 23722916 PMC3752543

[B18] Masuoka T. Kudo M. Yoshida J. Ishibashi T. Muramatsu I. Kato N.. (2016). Long-term activation of group I metabotropic glutamate receptors increases functional TRPV1-expressing neurons in mouse dorsal root ganglia. Front. Cell Neurosci. 10:79. 10.3389/fncel.2016.00079 27064319 PMC4814719

[B19] Masuoka T. Nakamura T. Kudo M. Yoshida J. Takaoka Y. Kato N.. (2015). Biphasic modulation by mGlu5 receptors of TRPV1-mediated intracellular calcium elevation in sensory neurons contributes to heat sensitivity. Br. J. Pharmacol. 172, 1020–1033. 10.1111/bph.12962 25297838 PMC4314192

[B20] Meng J. Wang J. Steinhoff M. Dolly J. O. (2016). TNFα induces co-trafficking of TRPV1/TRPA1 in VAMP1-containing vesicles to the plasmalemma via Munc18–1/syntaxin1/SNAP-25 mediated fusion. Sci. Rep. 6:21226. 10.1038/srep21226 26888187 PMC4758037

[B21] Motter A. L. Ahern G. P. (2012). TRPA1 is a polyunsaturated fatty acid sensor in mammals. PLoS ONE 7:e38439. 10.1371/journal.pone.0038439 22723860 PMC3378573

[B22] Ohta T. Imagawa T. Ito S. (2007). Novel agonistic action of mustard oil on recombinant and endogenous porcine transient receptor potential V1 (pTRPV1) channels. Biochem. Pharmacol. 73, 1646–1656. 10.1016/j.bcp.2007.01.029 17328867

[B23] Paulsen C. E. Armache J. P. Gao Y. Cheng Y. Julius D. (2015). Structure of the TRPA1 ion channel suggests regulatory mechanisms. Nature 520, 511–517. 10.1038/nature14367 25855297 PMC4409540

[B24] Rosenbaum T. Simon S. A. (2007). TRPV1 receptors and signal transduction in TRP Ion Channel Function in Sensory Transduction and Cellular Signaling Cascades, eds Liedtke W. B. Heller S. (Boca Raton, FL: CRC Press), 69–84. 21204486

[B25] Stanchev D. Blosa M. Milius D. Gerevich Z. Rubini P. Schmalzing G. (2009). Cross-inhibition between native and recombinant TRPV1 and P2X_3_ receptors. Pain 143, 26–36. 10.1016/j.pain.2009.01.006 19223122

[B26] Staruschenko A. Jeske N. A. Akopian A. N. (2010). Contribution of TRPV1-TRPA1 interaction to the single channel properties of the TRPA1 channel. J. Biol. Chem. 285, 15167–15177. 10.1074/jbc.M110.106153 20231274 PMC2865321

[B27] Szteyn K. Rowan M. P. Gomez R. Du J. Carlton S. M. Jeske N. A. (2015). A-kinase anchoring protein 79/150 coordinates metabotropic glutamate receptor sensitization of peripheral sensory neurons. Pain 156, 2364–2372. 10.1097/j.pain.0000000000000295 26172554 PMC4816074

[B28] Takahashi N. Mizuno Y. Kozai D. Yamamoto S. Kiyonaka S. Shibata T.. (2008). Molecular characterization of TRPA1 channel activation by cysteine-reactive inflammatory mediators. Channels 2, 287–298. 10.4161/chan.2.4.6745 18769139

[B29] Takayama Y. Uta D. Furue H. Tominaga M. (2015). Pain-enhancing mechanism through interaction between TRPV1 and anoctamin 1 in sensory neurons. Proc. Natl. Acad. Sci. U.S.A. 112, 5213–5218. 10.1073/pnas.1421507112 25848051 PMC4413337

[B30] Tang H. B. Inoue A. Oshita K. Nakata Y. (2004). Sensitization of vanilloid receptor 1 induced by bradykinin via the activation of second messenger signaling cascades in rat primary afferent neurons. Eur. J. Pharmacol. 498, 37–43. 10.1016/j.ejphar.2004.07.076 15363973

[B31] Terenghi G. Riveros-Moreno V. Hudson L. D. Ibrahim N. B. Polak J. M. (1993). Immunohistochemistry of nitric oxide synthase demonstrates immunoreactive neurons in spinal cord and dorsal root ganglia of man and rat. J. Neurol. Sci. 118, 34–37. 10.1016/0022-510X(93)90242-Q 7693875

[B32] Trevisani M. Smart D. Gunthorpe M. J. Tognetto M. Barbieri M. Campi B.. (2002). Ethanol elicits and potentiates nociceptor responses via the vanilloid receptor-1. Nat. Neurosci. 5, 546–551. 10.1038/nn852 11992116

[B33] Weng H. J. Patel K. N. Jeske N. A. Bierbower S. M. Zou W. Tiwari V.. (2015). Tmem100 is a regulator of TRPA1-TRPV1 complex and contributes to persistent pain. Neuron 85, 833–846. 10.1016/j.neuron.2014.12.065 25640077 PMC4336228

[B34] Wilson S. R. Gerhold K. A. Bifolck-Fisher A. Liu Q. Patel K. N. Dong X.. (2011). TRPA1 is required for histamine-independent, Mas-related G protein-coupled receptor-mediated itch. Nat. Neurosci. 14, 595–602. 10.1038/nn.2789 21460831 PMC3181150

[B35] Zurborg S. Yurgionas B. Jira J. A. Caspani O. Heppenstall P. A. (2007). Direct activation of the ion channel TRPA1 by Ca^2+^. Nat. Neurosci. 10, 277–279. 10.1038/nn1843 17259981

